# Outcome after kidney transplantation in hepatitis B surface antigen-positive patients

**DOI:** 10.1038/s41598-021-91331-y

**Published:** 2021-06-03

**Authors:** Hyejin Mo, Sangil Min, Ahram Han, In Mok Jung, Jongwon Ha

**Affiliations:** 1 Department of Surgery, Seoul Metropolitan Government-Seoul National University Boramae Medical Center, Seoul, South Korea; 2 Department of Surgery, Seoul National University College of Medicine, 101 Daehak-ro, Jongno-gu, Seoul, 03080 South Korea; 3 Transplantation Research Institute, Seoul National University College of Medicine, Seoul, South Korea

**Keywords:** Kidney, Viral hepatitis

## Abstract

Few reports detail the actual outcome of Hepatitis B Surface Antigen-positive patients after kidney transplant. HBsAg-positive patients who underwent kidney transplant between January, 1999, and December, 2018, were reviewed retrospectively. Outcomes including hepatitis B reactivation rate, risk factors for reactivation, and patient and graft survival rates were analyzed. Seventy-seven patients were enrolled (47.1 ± 11.5 years old). Patients received ABO-incompatible (n = 5), crossmatch positive transplant (n = 2), and re-transplant (n = 4). Forty-six patients received prophylactic; 19, medication at least 3 months before the transplant; and 12, did not receive medication. Seventeen out of 76 patients developed reactivation post-transplant. 52.9% of HBV reactivation was accompanied by hepatitis. Inappropriate, other than lifelong prophylactic, antiviral agents (HR = 7.34, 95% CI 1.51–35.69, *P* = 0.01) and high hepatitis DNA (≥ 1000 IU/ml) pre-transplant (HR = 4.39, 95% CI 1.08–17.81, *P* = 0.04) increased reactivation risk. There was no significant difference in patient and graft survival between antigen positive patients who received antiviral agent and propensity score matched negative patients. HBsAg positivity in kidney transplant recipients is associated with substantial HBV reactivation rate. Lifelong antiviral therapy is mandatory, and patients with high preop HBV titer should be monitored closely for HBV reactivation.

## Introduction

Before introduction of antiviral agents, infection with hepatitis B virus (HBV) is known to be associated with high mortality and morbidity in kidney transplant recipients^1,2^. Although the introduction of antiviral agents improved patient and graft survival, the risk of liver failure is still higher in HBV infected patients than uninfected patients^3^. HBV reactivation is one of the main reasons for liver failure in HBV infected patients and is known to be associated with a higher rate of liver failure compared to novel infection^4^. Therefore, several studies have been conducted to identify the risk factors of HBV reactivation in order to increase chances of successful transplantation^5–11^.

The current guideline recommends preemptive or prophylactic treatment for anti-HBc positive patients and prophylactic antiviral for Hepatitis B Surface Antigen (HBsAg) positive patients when they need immunosuppressive therapy for transplant^12^. Most of the evidence, however, is based on patients undergoing chemotherapy^13^. There are few reports about outcomes including HBV reactivation in HBsAg-positive patients in kidney transplant setting because of its rare incidence. The objective of this study is to investigate real-world outcomes including HBV reactivation after kidney transplantation in HBsAg-positive patients. We also aim to evaluate the safety of kidney transplants in HBsAg-positive patients.

## Results

### Patient characteristics

During study period, a total of 1968 patients received kidney transplantations at Seoul National University Hospital; Among these, 77 (3.9%) patients were HBsAg-positive at the time of transplantation. Baseline patient characteristics were summarized in Table 1. Thirty-five (45.5%) patients received organs from living donors. Sixty-five (84.4%) patients received basiliximab as induction therapy and 63 (81.8%) patients were maintained on a triple immunosuppressive regimen consisting of tacrolimus, mycophenolate mofetil, and prednisolone. Seventy-four patients had normal alanine aminotransferase (ALT) except for three patients with mild ALT elevation. HBV DNA was ≥ 1000 IU/ml in 20.8% of patients before transplantation. None of the patients were co-infected with hepatitis C virus.

**Table 1:** Baseline characteristic and immunosuppressants of 77 HBsAg positive kidney transplant recipients.

	Total (N = 77)
Male	59 (76.6)
Age (years)	47.1 ± 11.5
**Cause of ESRD**	
Glomerulonephritis	20 (26.0)
Diabetes	15 (19.5)
Hypertension	3 (3.9)
IgA	10 (13.0)
Polycystic kidney disease	2 (2.6)
Other	6 (7.8)
Unknown	21 (27.3)
Living donor	35 (45.5)
Re-transplantation	4 (5.2)
ABOi	5 (6.5)
HLA mismatches	2.8 ± 1.7
Positive crossmatch	2 (2.6)
Abnormal ALT levels before treatment	3 (3.9)
**HBV status of recipients**	
HBsAg positive	77 (100.0)
Unknown baseline HBV DNA	16 (20.8)
HBV DNA < 1000 IU/ml	45 (58.4)
HBV DNV ≥ 1000 IU/ml	16 (20.8)
**HBV status of donors**	
HBsAg positive	23 (29.9%)
Anti-HBc positive/HBsAg negative	21 (27.3%)
**Immunosuppressants**	
ATG as induction	3 (3.9)
Basiliximab as induction	65 (84.4)
Rituximab as desensitization	10 (13.0)
TAC + MMF + prednisolone as maintenance	63 (81.8)
CsA + MMF + prednisolone as maintenance	6 (7.8)
Other as maintenance	8 (10.4)

*HLA* human leukocyte antigen, *ATG* anti-thymocyte globulin.

Details of antiviral medication at the time of transplantation are depicted in Table 2. Of the 65 (84.4%) patients who took antiviral medication, 46 patients received prophylactic antiviral therapy. In the prophylactic group, 17 patients who received living kidney transplantation started medication between one week and the day before transplantation. Twenty-nine patients who received deceased donor kidney transplantation started prophylactic therapy on the day of transplantation. The remaining 19 patients were those who had been using antiviral medication over 3 months before the transplantation. More than half of these patients were administered entecavir (n = 47, 72.3%).

**Table 2:** Details of antiviral medications at the time of transplantation.

	Total (N = 77)
No medication, n (%)	12 (15.6)
**Prophylactic, n (%)**	46 (59.7)
Entecavir	37
Lamivudine	8
Telbivudine	1
**Antiviral therapy before transplant, n (%)**^**a**^	19 (24.7)
Entecavir	10
Lamivudine	3
Adefovir	3
Tenofovir	1
Combination therapy^b^	2

^a^At least 3 months before transplant.

^b^1 Adefovir + telbivudine and 1 adefovir + lamivudine.

### Incidence and risk factors for HBV reactivation

**Figure 1:**
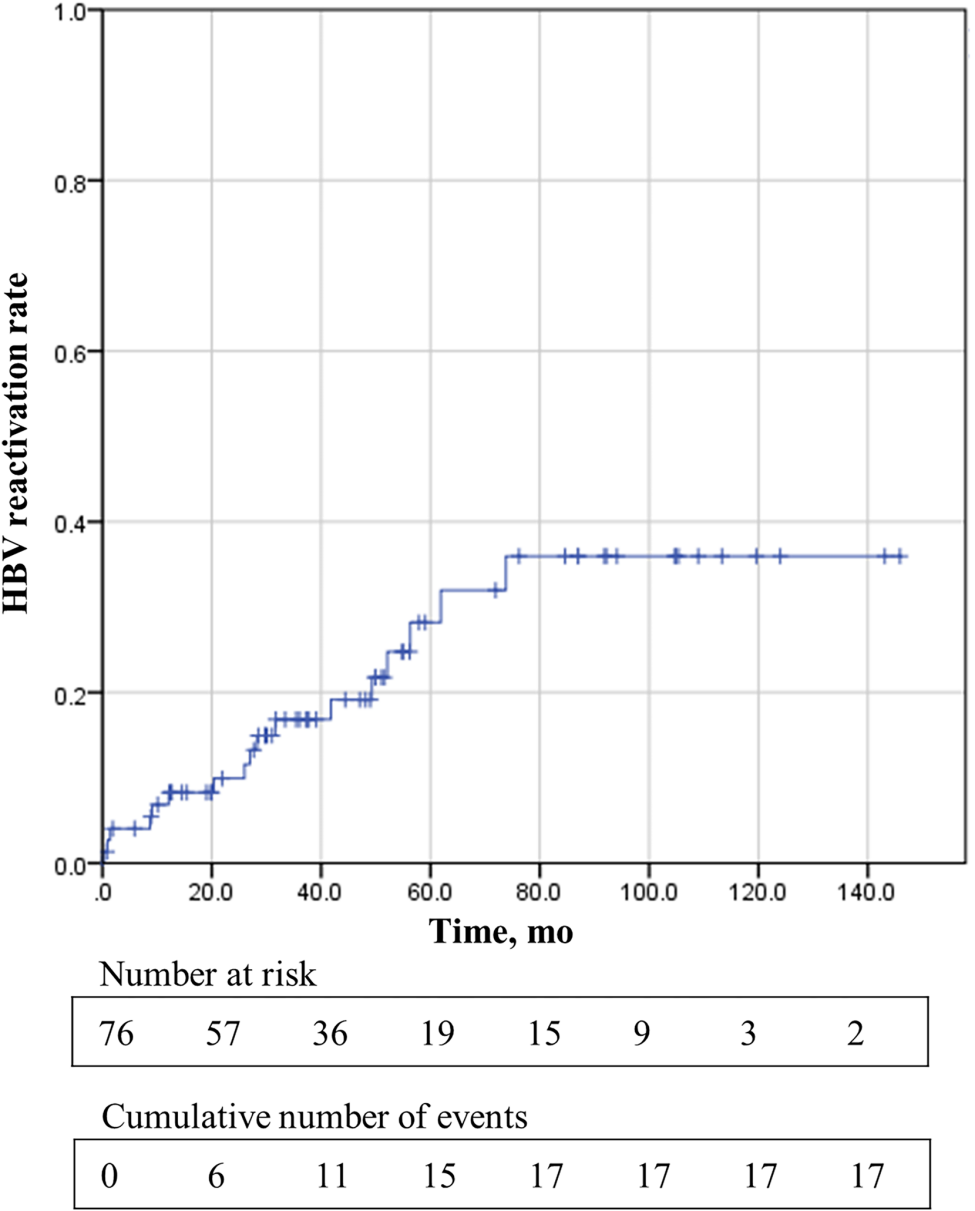
Kaplan–Meier curve for cumulative probability of HBV reactivation in 76 HBsAg positive recipients.

HBV DNA was checked in 76 patients after transplantation, except in one patient who received a transplantation in 1999. With mean follow up of 76.4 ± 48.5 months, HBV reactivation was noted in 17 patients (22.4%). The median time to reactivation was 27 months (range 0.3–73.8). 52.9% of HBV reactivation was accompanied by hepatitis. Kaplan–Meier curve for cumulative probability of HBV reactivation of 76 HBsAg-positive patients is shown in Fig. 1. Using Cox proportional hazards regression, inappropriate antiviral therapy—all except lifelong antiviral therapy—(HR = 7.34, 95% CI 1.51–35.69, P = 0.01) and HBV DNA higher than 1000 IU/ml before transplantation (HR = 4.39, 95% CI 1.08–17.81, P = 0.04) increased the risk HBV reactivation (Table 3). A total of 22 patients received inappropriate antiviral therapy, eight of them did not take medication at all, three did not receive prophylactic therapy properly, and the remaining 11 were discontinued antiviral medication.

**Table 3:** Risk factors for HBV reactivation.

Factors	Univariate analysis		Multivariate analysis	
	HR (95% CI)	P	HR (95% CI)	P
Recipient age > 60 years old	1.40 (0.32, 6.13)	0.66		
Donor age > 60 years old	2.16 (0.62, 7.57)	0.23		
Use of ATG^a^	0.04 (0.00, 131.42)	0.44		
Use of rituximab^a^	1.00 (0.23, 4.44)	0.99		
Acute rejection episodes	1.69 (0.63, 4.59)	0.30		
Inappropriate antiviral agent^b^	4.90 (1.73, 13.96)	0.00	7.34 (1.51, 35.69)	0.01
Preoperative HBV DNA titer ≥ 1000 IU/ml	5.54 (1.38, 22.15)	0.02	4.39 (1.08, 17.81)	0.04

*ATG* anti-thymocyte globulin.

^a^ATG and rituximab for both purposes of induction and anti-rejection treatment were included.

^b^Other than lifelong prophylactic antiviral therapy.

At the time of reactivation, 11 out of 17 patients (64.7%) were not on antiviral medication (5 patients did not take medication since transplantation, and six patients discontinued medication). Six patients experienced HBV reactivation while taking medication (lamivudine 3, entecavir 2, and adefovir 1). Of these, three patients who used lamivudine developed lamivudine resistance. Two patients who used entecavir and adefovir, respectively, had reactivation 12 months after transplantation. At that time, genetic tests showed no significant mutation and there was no evidence of noncompliance. The other one started prophylactic medication with entecavir, but reactivation occurred 1 week after transplantation.

### Patient and graft survival

**Figure 2:**
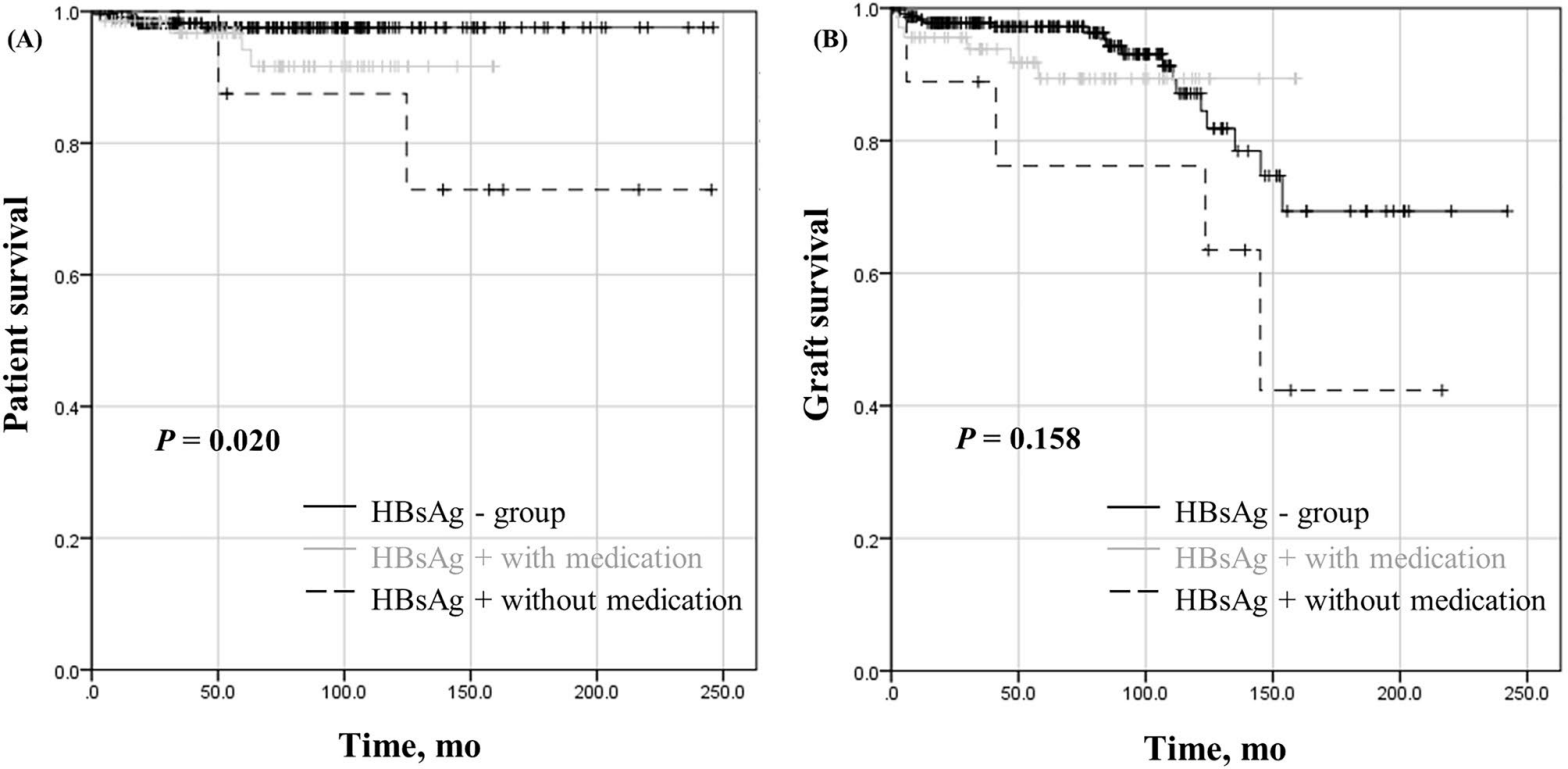
Kaplan–Meier curve for (**A**) patient survival and (**B**) graft survival.

Patients were propensity matched in a 1:3 (HBV positive: HBV negative) fashion with respect to covariates, which were age, sex, transplant year, type of donor, ABO incompatibility, and crossmatch positivity in this study. Patient and graft survival among three groups (HBsAg-positive patient who received antiviral mediation, HBsAg-positive patient who did not receive antiviral medication, and HBsAg-negative patients) was evaluated using Kaplan–Meier analysis and a log rank test. Figure 2 shows Kaplan–Meier survival curve for patient and graft survival. There was a significant difference in patient survival among the three groups (Fig. 2A, P = 0.020); However, between HBsAg-positive patients who received antiviral agent and HBsAg-negative patients there was no significant difference in patient survival. There was also no significant difference in death-censored graft survival among the three groups.

In the HBsAg-positive group, four patients died—four from liver failure, one from anaplastic astrocytoma in brain, and one from kidney failure. One of the four patients who died of liver failure was diagnosis with acute rejection 4 years after kidney transplantation. During treatment of rejection with steroids and OKT3, the patient died of liver failure caused by HBV reactivation. The second patient developed hepatocellular carcinoma 2 years after transplantation, and right after receiving segmental resection, was diagnosed with multiple hepatocellular carcinomas with bone metastasis. This patient underwent transcatheter arterial chemoembolization. However, 2 months after transcatheter arterial chemoembolization, liver failure gradually progressed and the patient died. Another patient underwent endoscopic sphincterotomy and endoscopic nasobiliary drainage due to the intrahepatic duct dilatation and subsequent hyperbilirubinemia. The patient was suspected of sclerosing cholangitis and was recommended for liver transplantation, however, died from sepsis and liver failure. The last patient was diagnosed with acute antibody-mediated rejection immediately after surgery and received therapy such as plasmapheresis and transfusion. Although the patient was HCV negative before surgery, during treatment the patient showed liver function test abnormality and HCV RNA was measured over 100,000,000 IU/ml. Later, he was treated with pegylated-interferon and ribavirin, but died from liver failure.

In the HBsAg-negative group five patients died—three from pneumonia, one from necrotizing fasciitis, and one from cerebral infarction. None of these deaths were related to liver failure.

## Discussion

To our knowledge, this is the largest study on the incidence and risk factors of HBV reactivation in HBsAg-positive kidney transplantation recipients. A total of 77 HBsAg-positive patients were analyzed, and 22.4% of them developed reactivation. Inappropriate antiviral agents and HBV DNA titer ≥ 1000 IU/ml before transplant increased the risk HBV reactivation. Eleven of the 17 (64.7%) patients were not on antiviral medication at the time of reactivation. With antiviral medication, there was no statistical difference in patient survival between HBsAg-negative patients and HBsAg-positive patients. There was no difference in death censored graft survival between HBsAg-negative and HBsAg-positive patients.

Before the introduction of antiviral therapy, HBV infection was considered to be a contraindication of kidney transplantations with mortality of up to 50%^1,2^. After introduction of lamivudine, HBV infection is effectively treated in kidney transplantation recipients^14–17^. Reddy PN et al. reported that in an era with the availability of oral anti-viral agents, patients and graft survival are not different between the HBsAg-positive and HBsAg-negative recipients^3^. This is consistent with the results of our study suggesting that with antiviral medications kidney transplantation can be safely carried out in HBsAg-positive patients.

In addition, Reddy PN et al. reported that acute liver failure is high in HBsAg-positive patients. HBV reactivation is one of the main reasons for liver failure in HBV infected patients, and liver failure is more likely to occur compared to novel infection^4^. Cytotoxic therapy and immunosuppression for solid organ transplantation are known as risk factors for HBV reactivation^5,6^. Most studies of HBV reactivation have been conducted in the setting of chemotherapy, however, some studies have also been done in the setting of transplantation. In a study of 322 occult HBV carriers, Chen GD et al. reported that older age (> 60 years) and using anti-T-cell antibodies are risk factors for HBV reactivation, and that patient survival is significantly lowered in the HBV reactivation group^7^. Lee J et al. reported results in 172 resolved HBV patients, and concluded that rituximab desensitization and the absence of anti-HBs antibodies were risk factors for HBV reactivation^9^. As reported by Kannan et al., in a study with 93 resolved HBV patients, the absence of anti-HBs antibodies is a risk factor for HBV reactivation and they underline that there is no HBV reactivation in patients with antibody titers greater than 100 IU/ml^11^. These studies have been done with anti-HBc positive patients. Due to the rare incidence of HBsAg-positive kidney recipients, there are few reports of HBV reactivation in HBsAg-positive patients. In our report, HBV reactivation is 22.4%, which is relatively high compared to 4.1–6.5% in the published literature of anti-HBc positive patients. This may indicate that HBsAg-positive patients need increased monitoring to detect HBV reactivation. In addition, considering that 64.7% (11/17) of reactivation occurs while not taking medication, lifelong prophylactic antiviral therapy is recommended.

Baseline high viremia is known to be a risk factor for HBV reactivation^18^. In the setting of liver transplantation, high HBV DNA titers (more than 10,000 copies/ml, approximately 2000 IU/ml) were identified as risk factors. However, this threshold value may be different because prophylaxis-using HBIG is not routinely injected in kidney transplantation patients^19^. Similarly, baseline HBV DNA of more than 2000 IU/ml was found to be a risk factor in the setting of cancer chemotherapy and other immunosuppressive drug therapies^20^. In our patients, the results showed that the risk of reactivation was high even with a titer of 1000 IU/ml. This suggests that close monitoring of HBV DNA is required in patients with high titers before kidney transplantation.

As shown in Table 1, because about 50% of patients received from donors with HBV history, we also evaluate whether donors’ HBV status impacts reactivation rate. Due to the risk of HBV transmission, donors with HBV are mostly matched to HBV recipients in Korea. In our study, 29.9% of donors were HBsAg-positive, and 27.3% were anti-HBc positive and HBsAg-negative. And there was no statistically significant difference in HBV reactivation according to donors’ HBV state (HBsAg+ vs. HBsAg− and anti-HBc+ vs. Anti-HBc−). However, to confirm whether this is a transmission or not, further studies using genotype are necessary.

Lamivudine is proven to be an effective treatment, but prolonged treatment with lamivudine is associated with a progressive increase in drug resistance^15–17^. Entecavir is one of the first line treatments because of its high resistance barrier and favorable safety profile^21^. Although there are only small studies about entecavir in renal transplant recipients, entecavir have been reported to be safe and efficient in HBV infected patients^22,23^. In our study, HBV reactivation occurred during medication use in six patients, with 5.4% (2/37) of patients using entecavir and 33.3% (3/9) of patients using lamivudine. Although a small number of cases precluded definitive conclusion, this may suggest that entecavir is better than lamivudine in reducing HBV reactivation.

The limitations of this report include its retrospective nature and the limited size of the population. Likely due to the small sample size, several factors known to be associated with HBV reactivation including older age (> 60 years), using anti-T-cell antibodies and rituximab were not significant in our study. Most patients were tested for HBV DNA annually and follow-up interval, and duration differed slightly from patient to patient. Therefore, it is possible that HBV reactivation is underestimated in our study. Data with regular follow-up in a large series is needed to assess the long-term effectiveness of antiviral therapy in HBsAg-positive patients. Finally, the heterogeneity of treatment is a limitation of this study. Immunosuppression is a life-long risk factor of reactivation, so antiviral agents should be used continuously^12^. In our study, prophylactic antiviral was discontinued in some of the patients who maintained antiviral therapy for more than 9 months and HBV DNA was negative. These decisions were made by individual physicians. In addition, most of the patients who were transplanted before the 2006s did not receive prophylactic antiviral medication. Even four out of 12 patients who did not receive antiviral medication had been transplanted after 2008, and it was difficult to know exactly why they did not receive medication. The heterogeneity of treatments made it difficult to draw definite conclusions.

Although HBsAg-positive patients are associated with substantial HBV reactivation rates, kidney transplantation can be safely carried out while taking antiviral agents to mitigate reactivation risk. Inappropriate antiviral medication and preoperative high HBV titer were found to increase the risk of HBV reactivation.

## Methods

### Patients

This was a retrospective observational study. Patients who received kidney transplantation at Seoul National University Hospital from January 1, 1999, to December 30, 2018, were reviewed. Patients who were HBsAg-positive at the time of transplant were included in the analysis. Patient information was collected retrospectively through medical chart review. Baseline characteristics of donors and recipients, viral status at the time of transplant, immunosuppressive regimen, and details of antiviral medication were collected. This study was conducted in accordance with the Declaration of Helsinki and was approved by the Seoul National University Hospital Institutional Review Board (1910-022-1067). Informed consent was waived for this study by the Seoul National University Hospital Institutional Review Board.

### Immunosuppressive protocol

Before 2008, it was not customary for patients to receive induction therapy. From 2008 on, patients received basiliximab or anti-thymocyte globulin (ATG) for induction therapy. For ABO incompatible kidney transplantation, patients received rituximab at a dose of 150 mg/m^2^ 1 week before plasmapheresis and underwent plasmapheresis until the isoagglutinin titers decreased to < 1:32. This was followed by administration of basiliximab for induction therapy. In cases of flow cytometric positive crossmatch, or donor specific HLA antibody positive transplantation, patients received rituximab at a dose of 375 mg/m^2^ and were induced with ATG. In most patients, maintenance immunosuppression consisted of tacrolimus, mycophenolate mofetil, and prednisolone. Tacrolimus was administered twice daily and adjusted to maintain a trough level of 8–12 ng/ml within the first 3 months, 6–8 ng/ml in 1 year, and 5–6 ng/ml after 1 year. Mycophenolate mofetil was administered at 0.5 g twice daily. Prednisolone was tapered to 5 mg daily after 1 month.

### Antiviral therapy

Before considering kidney transplantation, patients were given medication according to the guidelines for the management of HBV in the general population. As such, the group that had been on antiviral medication for 3 months prior to transplantation was included in the ‘antiviral therapy before transplant’ group. Except for these patients, patients who started antiviral therapy before receiving immunosuppressive therapy were included in the ‘prophylactic’ group. Patients who did not take medication at the time of transplantation were included in the ‘no medication’ group.

Lifelong prophylactic therapy is considered appropriate antiviral therapy, meaning taking the antiviral drug before starting immunosuppressants and maintaining lifelong treatment.

### Monitoring of HBV reactivation

In patients with a history of HBV infection, HBV DNA was measured before transplantation. HBV DNA was measured about 1-year interval after transplantation and when hepatitis occurred. HBV reactivation is defined as ≥ 2 log10 increase in HBV DNA levels from baseline, detection of HBV DNA with level > 100 IU/ml in a person with undetectable HBV DNA at baseline, or detection of HBV DNA with level ≥ 100,000 IU/ml in a person with no baseline.

We performed liver function tests daily during the first hospital admission for 2 weeks and at every visit thereafter. Patients followed up at the outpatient clinic every week for 1 month, every 2 weeks for the next month, monthly for 1 year, and after that every 2–3 months depending on their conditions. Hepatitis was defined as ≥ 3 three-times increase in serum ALT level that exceeded the reference range (> 58 IU/l) or ALT > 100 IU/l.

### Statistical analysis

All analyses were performed using the IBM SPSS Statistics/PC software package version 23.0 (IBM Corp., Armonk, USA). All normally distributed continuous variables are expressed as means and standard deviations. Categorical variables are reported as numbers and percentages. Cox proportional hazards regression was used to assess the effect of factors on HBV reactivation.

Patient and graft survival of HBsAg-positive patients were compared to that of propensity score matched HBsAg-negative patients. Propensity scores were calculated using covariates including age, sex, transplant year, type of donor, ABO incompatibility, and crossmatch positivity. HBsAg-positive patients were propensity matched in a 1:3 fashion with an HBsAg-negative recipient. Patient and graft survival were analyzed according to the Kaplan–Meier method, and the results were compared using the log-rank test. A p value < 0.05 was considered statistically significant.

## Abbreviations

ATG: Anti-thymocyte globulin
CI: Confidence interval
HBsAg: Hepatitis B surface antigen
HBV: Hepatitis B virus
HR: Hazard ratio
